# Cost-effectiveness analysis of sugemalimab vs. chemotherapy as first-line treatment of metastatic nonsquamous non-small cell lung cancer

**DOI:** 10.3389/fphar.2022.996914

**Published:** 2022-09-12

**Authors:** Zhiwei Zheng, Huide Zhu, Ling Fang, Hongfu Cai

**Affiliations:** ^1^ Department of Pharmacy, Cancer Hospital of Shantou University Medical College, Shantou, China; ^2^ Department of Pharmacy, Fujian Medical University Union Hospital, Fuzhou, China

**Keywords:** cost-effectiveness, nonsquamous non-small-cell lung cancer, sugemalimab, chemotherapy, first-line treatment

## Abstract

**Objective:** Sugemalimab is approved in China as a first-line treatment in combination with chemotherapy for metastatic nonsquamous non-small cell lung cancer (NSCLC). This study aims to evaluate the cost-effectiveness of first-line additional sugemalimab in combination with chemotherapy vs. chemotherapy from the perspective of the Chinese healthcare system.

**Materials and methods:** A three-state Markov model was designed to evaluate the costs and quality-adjusted life years (QALYs) of first-line sugemalimab combination with chemotherapy vs. chemotherapy over a 10-year period. Data on clinical outcomes were obtained from GEMSTONE-302 clinical trials. Costs and health utilities were collected from local databases and published literature. The uncertainty of the model parameters was explored through sensitivity analysis.

**Results:** Compared to chemotherapy, sugemalimab treatment for NSCLC resulted in an extra 0.50 QALYs at an additional cost of $73627.99, with an incremental cost-effectiveness ratio (ICER) of 148354.07/QALY at the willingness-to-pay (WTP) threshold of $37663.26/QALY. One-way sensitivity analysis indicated that the primary motivator in this model was the cost of sugemalimab. However, none of the parameters significantly affected the model’s results.

**Conclusion:** Sugemalimab combination therapy is not economically advantageous for the first-line management of metastatic non-squamous NSCLC, according to the Chinese healthcare system.

## Introduction

In 2020, there was estimated to be 19.3 million new cancer cases and nearly 10 million cancer deaths worldwide. Lung cancer is the leading cause of death among cancer deaths, causing an estimated 1.8 million deaths (18%) around the word (Sung et al., 2021). There were an estimated 870,982 new cases and 766,898 deaths in China, compared to 238,032 new cases and 144,913 deaths in the United States in 2022 (Xia et al., 2022). In China, there is an urgent need to face the enormous challenge of lung cancer prevention and treatment. Non-small cell lung cancers (NSCLC), which account for 80 to 90 percent of all primary lung cancers, are typically diagnosed as metastatic disease, a stage with a poor prognosis (Siegel et al., 2022). The overall 5 years survival rate of metastatic NSCLC was poor and reported as 10%–15% in China (Zheng et al., 2016). Clinical treatment guidelines recommended platinum-based chemotherapy as first-line chemotherapy and second-line chemotherapy (docetaxel, pemetrexed) as a subsequent treatment regimen before programmed cell death-1 (PD-1)/programmed cell death receptor ligand-1 (PD-L1) inhibitors were used in the treatment of metastatic NSCLC (Ettinger et al., 2022). However, it showed no advantage in terms of survival, with a median overall survival (OS) of 1 year (Ai et al., 2022).

In recent years, Several immune checkpoint inhibitors targeting PD-1 and PD-L1 have been suggested in guidelines and utilized in clinical practice (Paz-Ares et al., 2021; Sezer et al., 2021). In December 2021, sugemalimab was approved in China for the first-line treatment of epidermal growth factor receptor (EGFR) gene mutation and anaplastic lymphoma kinase (ALK) negative metastatic NSCLC administered in combination with pemetrexed and carboplatin for nonsquamous NSCLC (Dhillon and Duggan, 2022). Recently, GEMSTONE-302 evaluated the efficacy and safety of adding sugemalimab in combination with platinum-based chemotherapy compared to chemotherapy alone in people with metastatic non-squamous NSCLC (Zhou et al., 2022). This trial revealed that, compared to chemotherapy alone, the combination therapy significantly prolonged the median overall survival (OS) by 5.1 months (22.8 vs. 17.7 months) and increased progression-free survival (PFS) (8.3 vs. 4.8 months) for patients with metastatic nonsquamous NSCLC.

However, the high price of sugemalimab greatly raises the cost for cancer patients. Therefore, it is essential to assess the cost-effectiveness of sugemalimab treatments. The primary objective of our study was to evaluate the cost-effectiveness of additional sugemalimab compared with chemotherapy in first-line treatment of metastatic nonsquamous NSCLC patients from the perspective of the Chinese healthcare system.

## Material and methods

### Model structure

**FIGURE 1:**
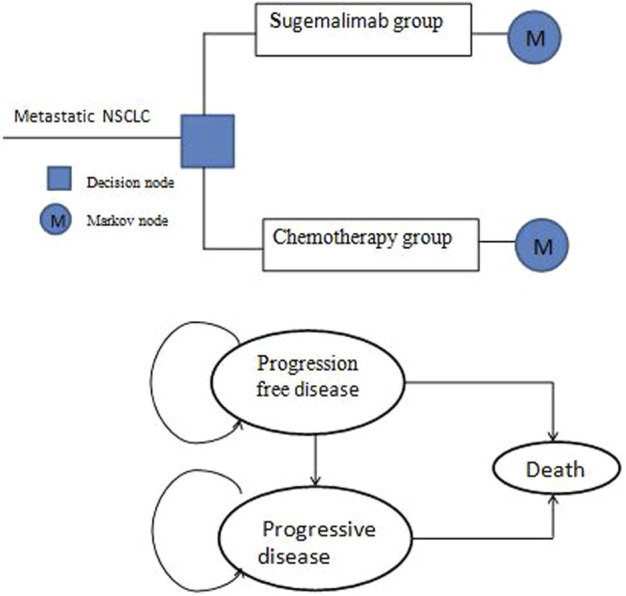
Model structure.

A three-partitioned survival structure provides the basis for our cost-effectiveness analysis (CEA) model. The model involves three distinct health states that represent different disease processes: progression-free disease (PFD), progressive disease (PD), and death (Figure 1). As patients received sugemalimab plus chemotherapy or chemotherapy alone once every 3 weeks in GEMSTONE-302 clinical trials, the model period was set to 3 weeks cycle length. The Markov model has a temporal span of 10 years. The 10-years time horizon was chosen since the general 5-years survival rate for metastatic NSCLC in China has been estimated to be 10%–15%.

The GetData Graph Digitizer 2.25 software was used to digitally extract survival data from the GEMSTONE-302 survival curves. The digitized R package (https://github.com/tpoisot/digitize/) was used to rebuild the Kaplan-Meier survival curves (Saluja et al., 2019). Survival probabilities were estimated using the Weibull, Log-logistic, Log-normal, Gompertz, Exponential, and Gamma distributions. The most appropriate distribution was then chosen based on visual examination and the values of the Akaike information criterion (AIC) and the Bayesian information criterion (BIC) (Bullement et al., 2019) (Supplementary Figures S1-S4, Supplementary Table S1). Finally, we used the Weibull distribution to simulate the PFS and OS curves for both situations. The Weibull rate as a function of survival over time was calculated using the formula using s(t) = exp (-λt^γ^) (Diaby et al., 2014). The estimated scale (λ),shape (γ) were presented in Table 1.

**TABLE 1:** Survival Estimates parameters.

Variable	Shape (γ)	Scale (λ)
weibull OS survival model		
Sugemalimab group	1.15	0.017
Chemotherapy group	1.36	0.015
weibull PFS survival model		
Sugemalimab group	1.16	0.047
chemotherapy group	1.29	0.059

We validated the models both internally with the GEMSTONE-302 trial and externally with published studies from the KEYNOTE-042 trial. The simulated PFS and OS curves closely resembled those given in the clinical trials, according to the internal validation. As part of the external validation, we compared the survival curves utilized in this study with those for the same therapy in other published studies. The PFS and OS curves were compared to those from the KEYNOTE-042 trial (Wu et al., 2021), which found similar 12-months OS rates.

The primary outcomes of the research were total life years, total costs, ICERs, andQALYs. Future costs and benefits were discounted at a rate of 5% according to the practice of pharmacoeconomic evaluation guidelines for universal health coverage in China (Yue et al., 2021). All expenses are presented in US dollars, with an average RMB translation rate of 6.45 RMB per US dollar (State Administration of Foreign Exchange, 2021). Additionally, the willingness-to-pay (WTP) was set at $37663.26/QALY in accordance with recommendations 3 times of 2021 gross domestic product (GDP) of China (National Bureau of statistics of China (2021); Yue et al., 2021). TreeAge Pro 2020 was used for the model and statistical analysis (Williamstown, MA).

### Patients and interventions

The medical data was obtained from the GEMSTONE-302 trial. The target population of this study is consistent with GEMSTONE-302 clinical trials. Patients with stage IV non-squamous NSCLC without known EGFR sensitising mutations, ALK, ROS1, or RET fusions, no previous systemic treatment for metastatic disease, regardless of PD-L1 expression, were randomly assigned to receive treatmnet. Sugemalimab group received sugemalimab (1,200 mg, intravenously) plus carboplatin (area under the concentration–time curve [AUC] 5 mg/ml per min) and pemetrexed (500 mg/m^2^),while chemotherapy group received placebo plus carboplatin (AUC = 5) and pemetrexed (500 mg/m^2^) intravenously on day 1 of every 3-weeks cycle, for up to four cycles, followed by maintenance treatment with pemetrexed plus either sugemalimab or placebo. Median duration of study treatment was 7.2 months (4.2–15.4 months) with sugemalimab and 4.6 months (2.8–6.9 months) with placebo (Zhou et al., 2022).

479 individuals were randomly assigned between 13 December 2018, and 15 May 2020, with 320 (67%) receiving sugemalimab and 159 (33%) receiving a placebo. The data cutoff date for the following parameters was 15 March 2021.241 (75%) of 320 sugemalimab patients and 147 (93%) of 159 placebo patients terminated their assigned medication, primarily due to illness progression (168 [53%] vs. 115 [72%]), whereas 79 (25%) of 320 sugemalimab patients and 12 (8%) of 159 placebo patients were still receiving treatment. 155 (48%) of 320 patients receiving sugemalimab experienced progression or death events, compared to 113 (71%), of 159 patients receiving placebo.

The improvement in progression-free survival was maintained at the prespecified progression-free survival final analysis (data cutoff 15 March 2021), with disease progression or death events occurring in 223 (70%) of 320 patients treated with sugemalimab and 135 (85%) of 159 patients treated with placebo.

The grade 3 or 4 adverse events were chosen from the GEMSTONE-302 trial based on two criteria: 1) More than 10% of grade 3 or 4 adverse events occurred in the sugemalimab or chemotherapy groups; 2) the difference between the two groups was greater than 1%.

According to the GEMSTONE-302 trial, the proportion of patients who received at least one subsequent anticancer therapy was 44% in the sugemalimab group and 62% in the chemotherapy group. After communication and discussion with clinicians, it was concluded that the second-line treatment option after disease progression was a platinum plus docetaxel regimen. Following the failure of second-line treatment, the optimum third-line treatment was not defined, and the individual scheme was not shown in the GEMSTONE-302 trial. As a result, the therapy following progression was thought to be the best support treatment.

### Cost and utility estimates

Only direct medical care costs were covered in this model, including drug costs, the cost of serious adverse events (SAEs) management, follow-up costs, subsequent costs, best supportive care costs. The data sources of follow-up cost, subsequent cost, and BSC cost was collected from published literature. Drug prices were sourced from China’s health industry data platform (https:// www.yaozh.com/) in 2022. The chemotherapy dose was calculated based on a model of body surface area (1.72 m^2^, 65 kg) and creatinine clearance of 70 ml/min (Lu et al., 2017).

Utility values were used to reflect the effect of disease on health status and were evaluated by the patient’s preference for living in a certain health condition, with 0 being the worst and one representing the best (Cheng et al., 2021). There is no data in the GEMSTONE-302 clinical study on the value of the PFS and PD. As a result, health status utilities were gathered from previous literature. The utility values for PFS, PD, and death were 0.71, 0.67, and 0, respectively (Nafees et al., 2017). The model also evaluated the disutility of the SAEs. All cost and utility values are displayed in Table 2.

**TABLE 2:** Model economic parameters and the range of the sensitivity analysis.

Variable	Baseline value	Range		Distribution	Source
		Minimum	Maximum		
Drug cost (US dollar $)					
Sugemalimab per mg	3.08	1.54	3.08	Gamma	YaoZH (2022)
pemetrexed per mg	1.672	1.337	2.006	Gamma	YaoZH (2022)
Carboplatin per mg	0.041	0.032	0.049	Gamma	YaoZH (2022)
Costs of serious adverse events per cycle ($)					
Anemia	531.7	425.36	638.04	Gamma	Yang et al. (2021)
White blood cell count decreased	461.5	369.2	553.08	Gamma	Yang et al. (2021)
Platelet count decreased	3,551.7	2,841.36	4,246.04	Gamma	Yang et al. (2021)
Sugemalimab group AEs (grade≥3) incidence (%)					
Anemia	0.13	—	—	Gamma	Zhou et al. (2022)
White blood cell count decreased	0.14	—	—	Gamma	Zhou et al. (2022)
Platelet count decreased	0.11	—	—	Gamma	Zhou et al. (2022)
chemotherapy group AEs (grade ≥3) incidence (%)					
Anemia	0.69	—	—	Gamma	Zhou et al. (2022)
White blood cell count decreased	0.17	—	—	Gamma	Zhou et al. (2022)
Platelet count decreased	0.10	—	—	Gamma	Zhou et al. (2022)
Utility value					
Progression-free disease	0.71	0.53	0.89	Beta	Nafees et al. (2017)
Progressive disease	0.67	0.50	0.84	Beta	Nafees et al. (2017)
Disutility due to AEs					
Anemia	0.073	0.058	0.088	Beta	Nafees, et al. (2017)
White blood cell count decreased	0.2	0.16	0.24	Beta	Nafees, et al. (2017)
Platelet count decreased	0.108	0.086	0.13	Beta	Zhou et al. (2019)
Other					
Follow-up cost per cycle	55.60	27.8	83.4	Gamma	Li et al. (2019)
Subsequent therapy cost per cycle	854.05	427.02	1,281.08	Gamma	Luo et al. (2022)
Best supportive care per cycle	337.50	168.75	506.25	Gamma	Qiao et al. (2021)
Patient weight (kg)	65	32.5	97.5	Normal	Lu et al. (2017)
Body surface area (m2)	1.72	0.86	2.58	Normal	Lu, et al. (2017)
Creatinine clearance rate (ml/min)	70	35	105	Normal	Lu et al. (2017)
Discount rate (%)	5	0	8	Beta	Yue et al. (2021)

### Sensitivity analysis

We conducted one-way sensitivity and probabilistic sensitivity analyses to evaluate the key factors that influence cost-effectiveness. In the one-way sensitivity analysis, the effect of different parameters on ICER was altered to a range of ±25% of the base case value, with the exception of the current price of sugemalimab, which fluctuated by 50%.

A Probabilistic sensitivity analysis (PSA) was carried out by running 10,000 Monte Carlo simulations to test the uncertainty of the model with all parameters simultaneously varied within a specific pattern of distribution, in which costs were given to a gamma distribution, probability parameters were given to a beta distribution, and utilities were given to a beta distribution (Haji Ali Afzali et al., 2018). The PSA results were presented in the form of scatter plots and cost-effectiveness acceptability curves.

## Results

### Base case results

In comparison with chemotherapy, Sugemalimab group produced an incremental 1.74 expected overall life years and 1.160 QALYs, with an incremental cost of $73627.99, which led to an ICER of $148354.07/QALY at the threshold of $37663.26/QALY in the China (Table 3).

**TABLE 3:** The base results of the cost-effectiveness analysis.

Treatment	Total cost ($)	Total life years	Total QALYs	Incremental cost ($)	Incremental QALY	ICER ($/QALY)
Sugemalimab group	98531.06	1.74	1.16	73627.99	0.50	148354.07
Chemotherapy group	24903.07	1.03	0.66	—	—	—

ICER: Incremental cost–effectiveness ratio; QALY: Quality-adjusted life year.

### Sensitivity analyses

**FIGURE 2:**
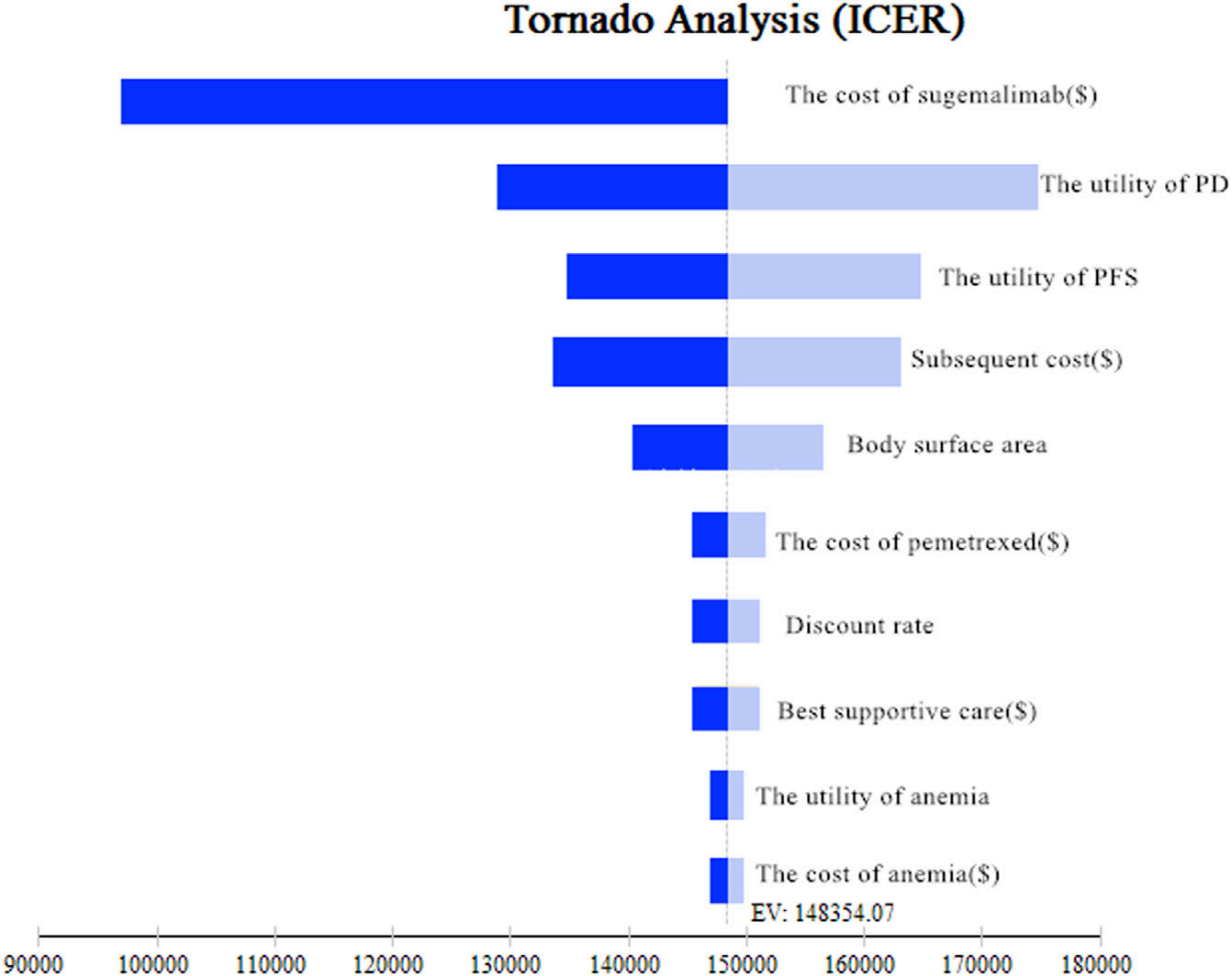
Tornado diagram of one-way sensitivity analyses of sugemalimab vs. chemotherapy.

The results of the one-way sensitivity analysis were shown in the Tornado diagram (Figure 2), which indicated that the cost of sugemalimab had the most effect on model outcomes in China. When its value was down from 0 to 50%, the ICERs of sugemalimab adjusted from $148354.07/QALY to $97608.56/QALY, respectively. However, it is still above the WTP and has not affected the model’s results.

The model was also very sensitive to the utility of PD, the utility of PFS, the cost of subsequent treatment, body surface, the cost of pemetrexed, discount rate, the cost of best supportive care, the utility of anemia, the cost of anemia. However, none of the variables had a substantial impact on the model’s output.

**FIGURE 3:**
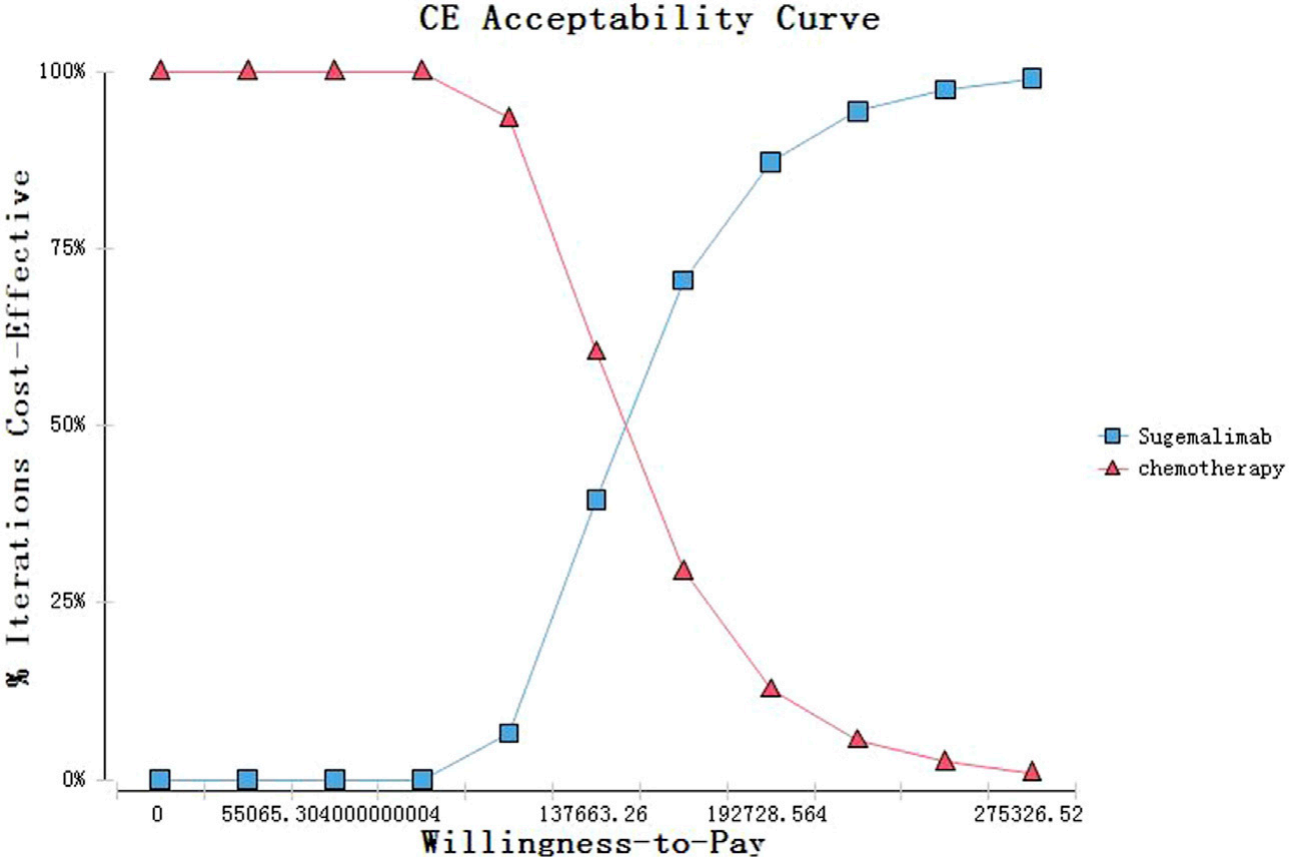
Cost-effectiveness acceptability curves of sugemalimab vs chemotherapy.

The cost-effectiveness acceptability curves showed a nearly 0% probability of Sugemalimab group and a 100% probability of chemotherapy being a cost-effective strategy at the threshold of $37663.26/QALY in China (Figure 3).

## Discussion

Due to the rising incidence and death of lung cancer, oncologists and patients are interested in the GEMSTONE-302 clinical trial benefits of sugemalimab. But the high cost of anti-cancer drugs could limit their widespread use. Estimating the cost-effectiveness of a novel treatment is a crucial prerequisite before offering patients access to the treatment regimen given the disease and economic burden of advanced NSCLC. Yang et al. (2021) compared the cost-effectiveness of atezolizumab in combination with carboplatin and nabpaclitaxel-based chemotherapy to chemotherapy alone in advanced NSCLS patients. They discovered that the atezolizumab combination is not cost-effective for first-line treatment from the Chinese healthcare system perspective. When compared to chemotherapy, cemiplimab was reported by Zhang M et al. (2022) to be more cost-effective in the first-line treatment of non-small cell lung cancer (NSCLC) in patients who are at least 50% PD-L1 positive.

The health system needs to control the irrational growth of health expenses and reduce residents’ overuse of health services (Wang et al., 2021). Our research addresses this emerging requirement for a sugemalimab economic evaluation.

Based on the results of the GEMSTONE-302 clinical trial,our analysis demonstrated sugemalimab plus chemotherapy for advanced nonsquamous NSCLC produced 1.160 QALYs, with an incremental cost of $73627.99, which led to an ICER of $148354.07/QALY at the threshold of $37663.26/QALY in the China. Sugemalimab combination therapy is not economically advantageous for the first-line management of advanced nonsquamous NSCLC, according to the Chinese healthcare system. The results of both the one-way and probabilistic sensitivity studies demonstrate that this result is generally robust.

We would caution readers not to consider this data as a reason to avoid using sugemalimab. We think that cost-effectiveness evaluations in cancer treatment should not be taken as evidence to limit the use of effective therapy, but rather as a tool to guide the development of scientific and reasonable prices for drugs and develop a medical insurance drug catalog.

The influence of medication pricing on cancer treatment options is one of the major issues raised by this study. This is especially true in China, where nonsquamous NSCLC accounts for more than one-third of all newly diagnosed cases of lung cancer globally (Cao et al., 2021). Hence, even a little rise in medicine prices might have a huge impact on the healthcare system (Ocran Mattila et al., 2021). A number of strategies have been taken by the Chinese government in recent years to lower the market price of anticancer medications in response to these challenges, including assistance for regional anticancer drug research and national drug price negotiations with suppliers (Tang et al., 2020). The price of several expensive anticancer treatments has been decreased by more than 50% as a consequence of the Chinese government’s implementation of many rounds of drug price talks with pharmaceutical producers since 2016 (Zhang Y et al., 2022).

This study has several strengths that should be highlighted. First and foremost, this is the first study in China to evaluate the cost-effectiveness of sugammalizumab in the treatment of metastatic non-squamous NSCLC. We selected reliable data, particularly PFS and OS statistics, from the most recent randomized phase 3 study, GEMSTONE-302. Second, the study complied with the Integrated Reporting Criteria for Health Economic Evaluation’s disclosure standards (Husereau et al., 2022). The study perspective, time range, hypotheses, and sources of validity evaluation were all well described, as well as the Patient characteristics of the base case. However, there are several limitations in this analysis. First, the clinical data in this study was obtained from the GEMSTONE-302 trial, but the OS curves of the patients in this trial have not yet reached the long-term follow-up results, which may affect the simulation of the long-term survival curves and increase the uncertainty of the calculated results. Hence, we set the simulation period to 10 years in this study based on the actual situation to reduce this bias. Second, Only SAEs in grades 3/4 were included in the study. We hypothesized that grade 1/2 SAEs would not affect the study’s final conclusion, and sensitivity analysis showed that the result was unchanged by factors related to SAEs. Thirdly, in the real situation, the subsequent treatment plan of patients may vary due to the individual. We only adopted the recommendations of platinum-based plus docetaxel or pemetrexed in the Chinese Society of Clinical Oncology (CSCO) guidelines for the treatment of non-small cell lung cancer (Chinese Association for Clinical Oncologists and Medical Oncology Branch of Chinese International Exchange, 2021), without considering individual differences. Sensitivity analysis showed that the result was unchanged by factors related to subsequent treatment.

Despite the limitations of this study, the uncertainty analysis was explored in detail and demonstrated the robustness of the underlying analysis results, so the results and conclusions of this study remain robust for clinical treatment decisions and health insurance access negotiations.

## Conclusion

From the perspective of China’s healthcare payers, sugemalimab plus chemotherapy is expensive and not cost-effective for patients with metastatic non-squamous NSCLC.

## Data Availability

The raw data supporting the conclusions of this article will be made available by the authors, without undue reservation.

## Supplementary material

Supplementary Figures S1 Modes simulation visual overall survival curve of sugemalimab group.

Supplementary Figures S2 Modes simulation visual overall survival curve of chemotherapy group.

Supplementary Figure S3 Modes simulation visual progression-free survival curve of sugemalimab group.

Supplementary Figure S4 Modes simulation visual progression-free survival curve of chemotherapy group.

Supplementary Table S1 Comparison of survival models.
