# Supplementary figures and images for: Cost-effectiveness analysis of sugemalimab vs. chemotherapy as first-line treatment of metastatic nonsquamous non-small cell lung cancer
Source: Front Pharmacol. 2022 Sep 12;13:996914. doi: 10.3389/fphar.2022.996914 (PMC9511109; doi:10.3389/fphar.2022.996914)

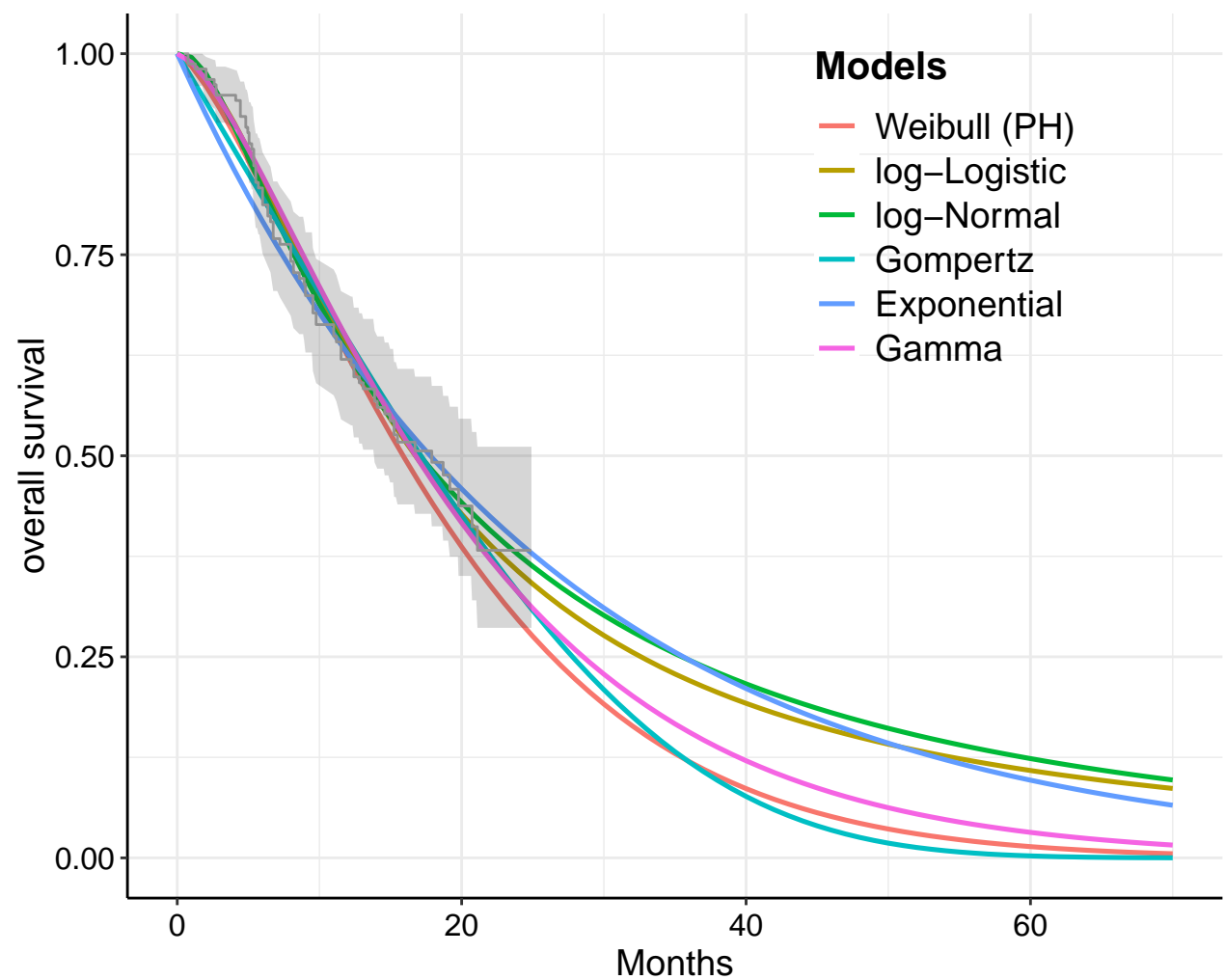

Supplement: Supplementary file 1 [file DataSheet2.PDF]

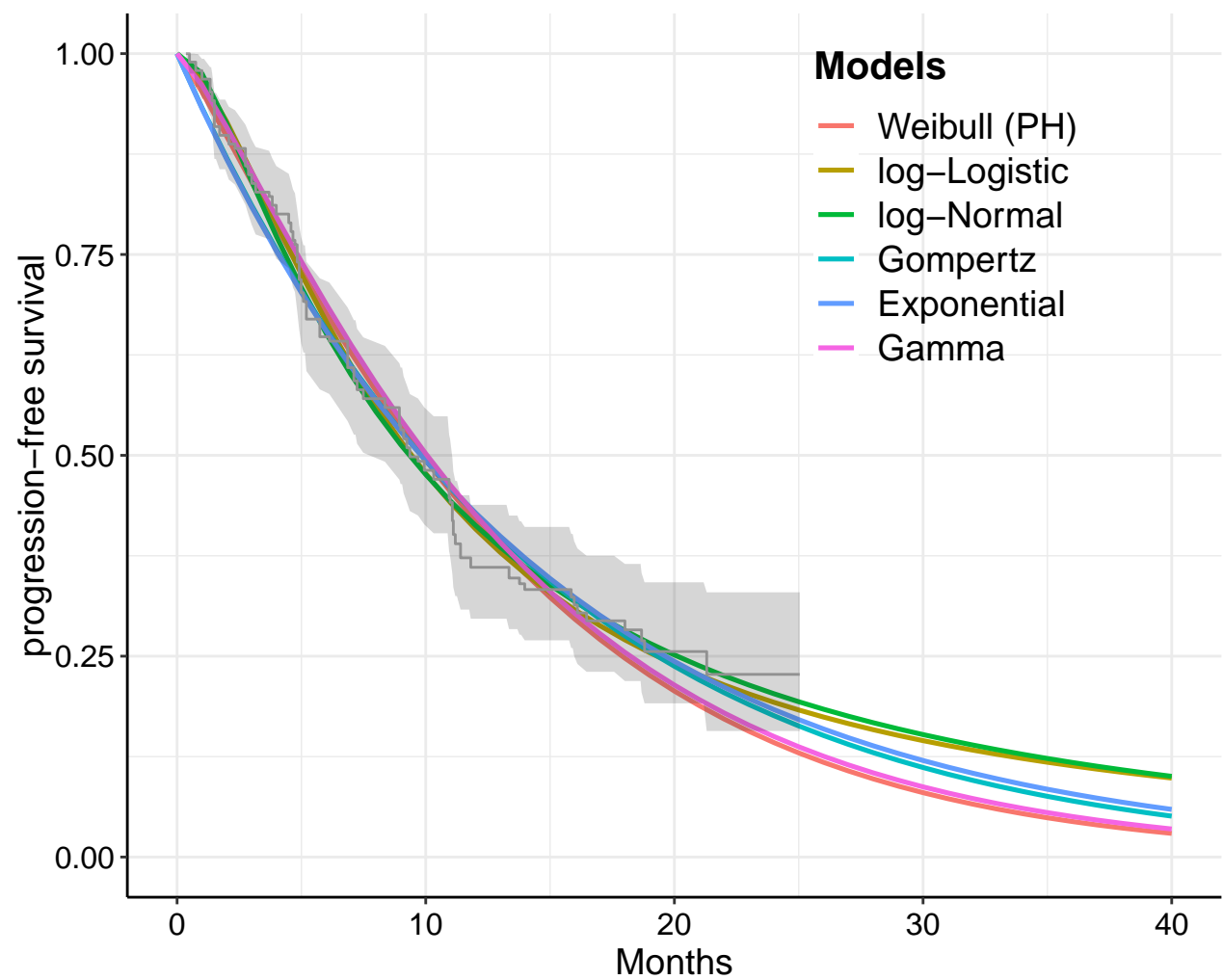

Supplement: Supplementary file 2 [file DataSheet4.PDF]

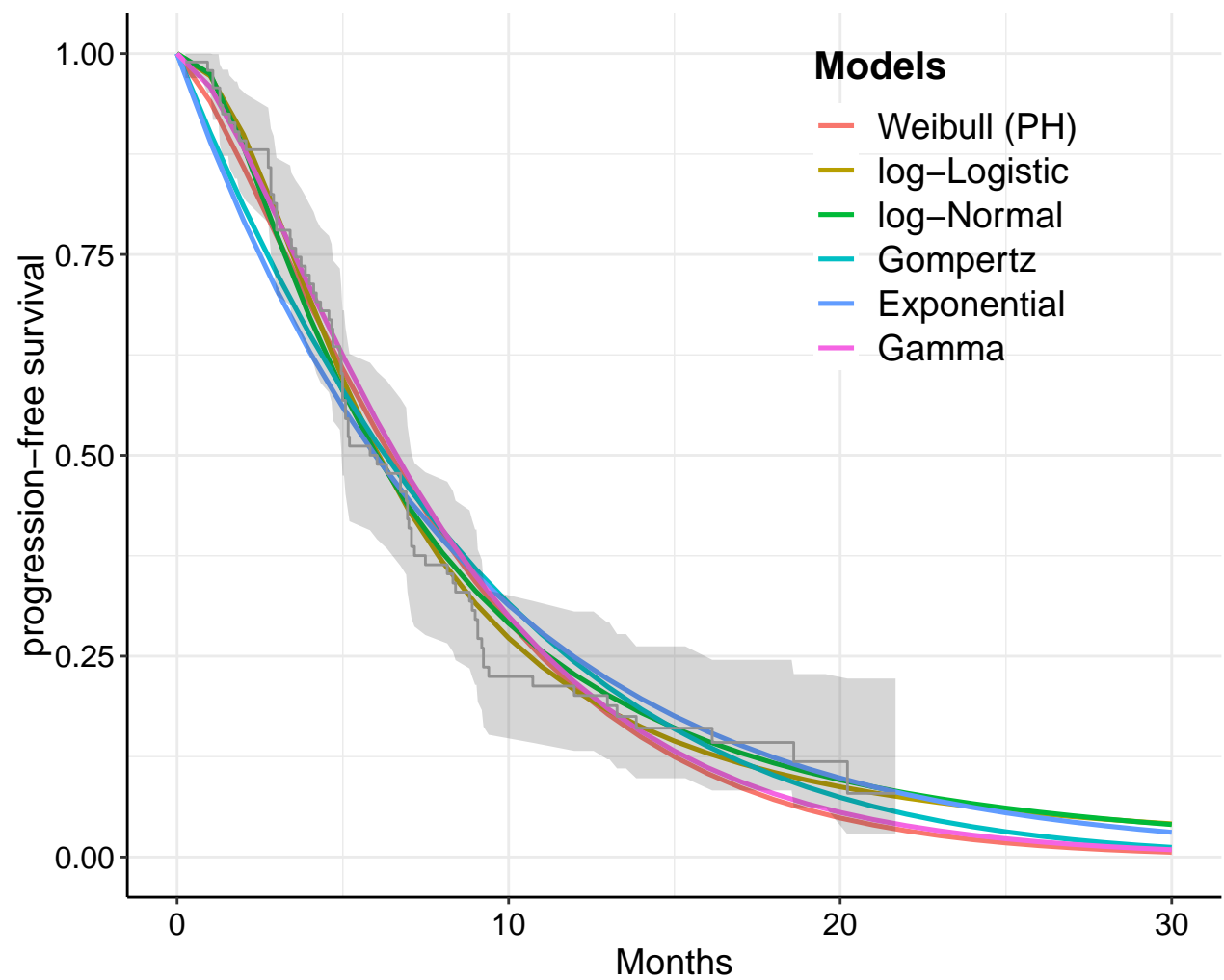

Supplement: Supplementary file 4 [file DataSheet3.PDF]

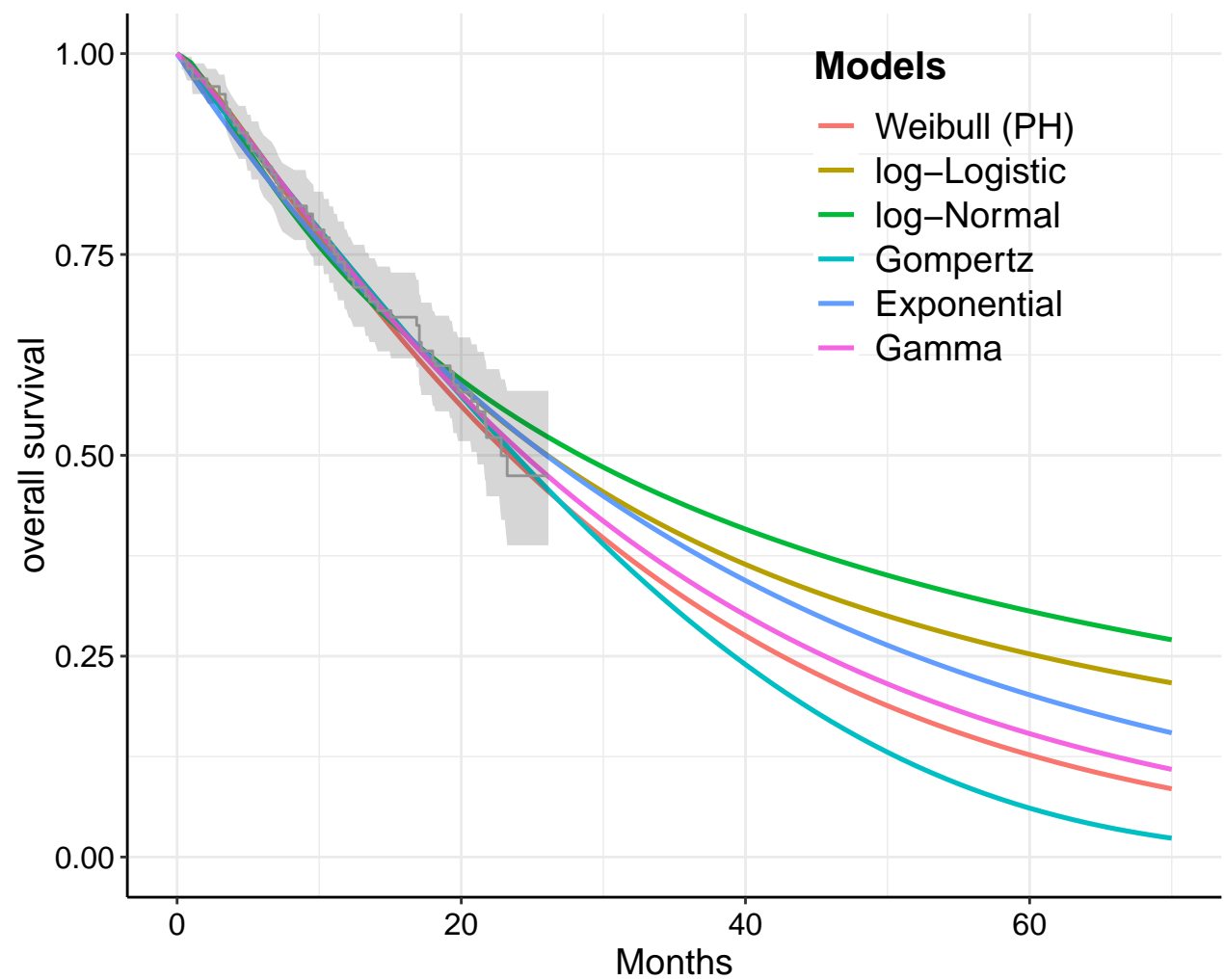

Supplement: Supplementary file 5 [file DataSheet1.PDF]
