# Supplementary material for: Cost-effectiveness analysis of sugemalimab vs. chemotherapy as first-line treatment of metastatic nonsquamous non-small cell lung cancer
Source: Front Pharmacol. 2022 Sep 12;13:996914. doi: 10.3389/fphar.2022.996914 (PMC9511109; doi:10.3389/fphar.2022.996914)
Supplement: Supplementary file 3 [file Table1.DOCX]

**Table S1. Comparison of survival models**

|  | AIC | | BIC | |
| --- | --- | --- | --- | --- |
|  | sugemalimab group | Chemotherapy group | sugemalimab group | Chemotherapy |
| PFS |  |  |  |  |
| **Weibull** | **935.8595** | **483.5692** | **942.3640** | **488.6979** |
| Log-logistic | 928.2654 | 474.3916 | 934.7699 | 479.5203 |
| Log-normal | 927.5185 | 477.7088 | 934.0231 | 482.8375 |
| Gompertz | 939.3889 | 489.9770 | 945.8934 | 495.1057 |
| Exponential | 937.4327 | 488.9136 | 940.6850 | 491.4780 |
| Gamma | 940.6850 | 480.6244 | 940.5653 | 485.7531 |
| OS |  |  |  |  |
| **Weibull** | **1111.982** | **633.5702** | **1119.518** | **639.7080** |
| Log-logistic | 1112.710 | 630.3331 | 1120.247 | 636.4709 |
| Log-normal | 1117.395 | 630.0670 | 1124.932 | 636.2048 |
| Gompertz | 1112.696 | 638.8923 | 1120.233 | 645.0301 |
| Exponential | 1112.772 | 639.8497 | 1116.540 | 642.9186 |
| Gamma | 1111.970 | 632.1425 | 1119.506 | 638.2804 |

AIC: Akaike information criterion; BIC: Bayesian Information Criterion; OS: Overall survival; PFS: Progression-free survival;
